# An experimental investigation of the influence of deviant peers on own deviancy: A replication study

**DOI:** 10.1007/s11292-017-9305-3

**Published:** 2017-10-01

**Authors:** N. Mercer, E. Crocetti, W. Meeus, S. Branje

**Affiliations:** 1 Research Centre Adolescent Development, Utrecht University, P.O. Box 80125, 3508 TC, Utrecht, The Netherlands; 2 Department of Psychology, Alma Mater Studiorum University of Bologna, Viale Berti Pichat 5, 40126 Bologna (BO), Italy; 3 Developmental Psychology, School of Social and Behavioral Sciences, Tilburg University, P.O. Box 90153, 5000 LE Tilburg, The Netherlands

**Keywords:** Causality, Delinquency, Deviancy, Experiment, Peer delinquency

## Abstract

**Objectives:**

This study is a replication of a study examining the causal impact of a brief exposure to deviant peers on own deviant behavior, i.e., Paternoster et al. (Journal of Research in Crime and Delinquency, 50:476–503, 2013). This study retested this design using different monetary incentives and a female deviant peer.

**Methods:**

A total of 69 university students (61% female) from the Netherlands participated in this laboratory-based study (Mage = 20.64; SD = 2.00) under the façade of a study on individual differences predicting memory recall. Participants could earn up to 10 euros. All participants had the opportunity to cheat to illegitimately earn more money (deviancy). Participants in the experimental condition were exposed to a deviant peer who verbalized her intention to cheat, justified this behavior, and then visibly cheated on the memory recall task.

**Results:**

Although participants in both conditions engaged in some deviancy, the brief exposure to a deviant peer significantly increased the amount of deviancy compared to participants who were not exposed to a deviant peer. These results were consistent after controlling for different demographic and theoretical control variables that predict deviancy.

**Conclusions:**

Although not identical in magnitude, our results echo those found by Paternoster et al. (2013): Even a brief exposure to a previously unknown deviant peer increases the amount of deviant behavior in young adults. Future research should examine factors predicting the susceptibility to (different types and thresholds of) deviant peer influence.

**Electronic supplementary material:**

The online version of this article (10.1007/s11292-017-9305-3) contains supplementary material, which is available to authorized users.

## Introduction

Undoubtedly, peer deviance is one of the most robust predictors of one’s own deviance (Pratt et al. 2010). Indeed, social learning theory (Akers 1998) and differential association theory (Sutherland 1947) suggest that people learn about criminal behavior by interacting with deviant others. Researchers have studied the role of deviant peers and the mechanisms by which they have influence for decades (e.g., Agnew 1991; Warr and Stafford 1991). But much of research in support of these prominent criminological theories is correlational, based on self-reported own deviancy and perceived peer deviancy, and is consequently confounded by the possibility of selection effects and projection bias. Therefore, this study aimed to contribute to the small body of experimental research on the influence of deviant peers by replicating previous work that examined the causal effect of a brief exposure to a deviant peer on own deviancy (i.e., Paternoster et al. 2013).

## The need for experimental studies of peer deviancy

While the relation between (perceived) peer deviancy and own deviancy is a robust, well-established criminological finding (e.g., Pratt et al. 2010), the meaning of this relation has been subject to much debate due to numerous methodological and theoretical challenges. First, control theorists have argued that the relation between peer delinquency and own delinquency is simply a matter of selection—people prone to delinquency are more likely to associate with other delinquents (e.g., Gottfredson and Hirschi 1990). Although longitudinal studies can provide evidence for the direction of effects from delinquency to deviant peers or vice versa, these studies cannot fully account for plausible selection effects related to, for example, any underlying similarities in criminal propensity.

Second is the issue of the measurement of peer delinquency. While much research conducted on deviant peers has used perceived measures of peer delinquency (e.g., Pratt et al. 2010), recent research has found that these perceptions are more related to self-projection and bias than to actual peer delinquency (e.g., Young and Weerman 2013). Not surprisingly, own delinquency seems to be more strongly correlated with perceived peer delinquency than with actual peer delinquency (Weerman and Smeek 2005).

Recent methodological advances such as social network analyses are coming closer to solving these on-going debates (e.g., Veenstra and Dijkstra 2011); however, experimental studies can, nevertheless, supplement the current state of research by already addressing some of these methodological challenges. While experimental studies are not without their own limitations (e.g., external validity), they can eliminate selection effects and projection bias. Further, random assignment should ensure that the characteristics of those exposed to deviant peers do not differ from those who are not, and manipulating peer deviancy and its mechanism of influence can shed light on the exact process(es) by which exposure to deviant peers influences own behavior (e.g., behavioral modeling, positive reinforcement, changes in routine activities).

## Previous experimental research on peer deviancy

Only a few studies to date have directly examined the role of deviant peers on own deviancy using an experimental design (Gallupe et al. 2016; Paternoster et al. 2013). While the experimental social psychology literature has a multitude of studies that examine peer influence on various behavioral outcomes (see Paternoster et al. 2013 for an overview), the outcomes of interest are often not comparable to (minor) delinquent acts. Similarly, developmental research has also conducted studies on the influence of (deviant) peers on risk-taking behavior or risky decision-making behavior (e.g., Gardner and Steinberg 2005; MacLean et al. 2014; Smith et al. 2014; van Hoorn et al. 2016). However, these studies manipulated either the presence of peers or their risk-aversive attitudes, but not their actual deviant behavior.

In the study by Paternoster et al. (2013), participants were told they could earn 1 dollar for every correct word on a memory recall task, up to a maximum of 20 dollars. All participants were given an opportunity to illegitimately earn this money by clicking on links in which the memory recall words were revealed. In the experimental condition, participants were exposed to a deviant peer who justified this illegitimate use of the links, verbalized his intention to use them, and then modeled deviancy by openly clicking on all of the links to unjustly “recall” more words. Further, Gallupe et al. (2016) used a similar design but increased the seriousness of the outcome by providing an opportunity to steal a 15-dollar gift card. Both studies found that exposure to a deviant peer led to deviancy. Given that these studies are among the first laboratory-based experimental studies to examine the influence of deviant peer modeling in criminology, replication and generalizability tests are warranted (see also Open Science Collaboration 2015).

## The current study

The aim of this study was to replicate the effect of a brief exposure to a deviant peer found in Paternoster et al. (2013), using the same design in a different sample. Further, in our study, there were small methodological differences: a different monetary award for deviancy, a female experimenter, and a female deviant peer. These differences test the replicability and generalizability of a brief exposure of deviant peers to contexts and characteristics beyond those of the original study.

## Method

### Participants

A total of 69 students (61% female; 91% Dutch) were recruited from Utrecht University (88%) and the HU University of Applied Sciences located in the Netherlands (M_age_ = 20.64; SD = 2.00; range 18–26). Participants were recruited for a study on “individual differences in memory recall” using posters located across campuses, online advertisements, and flyer handouts. The study took place in a 22-person computer lab at Utrecht University on one day in April 2015. There were six sessions, with a minimum of six and a maximum of 16 participants in each session. Participants signed up for a specific time slot on the basis of their availability and these time slots were randomly assigned to be three experimental (*n* = 33) and three control (*n* = 36) conditions. Only the lead researcher and the peer confederate knew which sessions were which to avoid any accidental bias from the experimenter. Informed consent was obtained from all participants. The Utrecht University Faculty of Social Sciences Ethical Committee approved this study.

### Procedure

This procedure is a replication of Paternoster et al. (2013), with some methodological differences: a different monetary reward, a female experimenter, and a female deviant peer. A detailed description of the full procedure can be found in the Technical Appendix.

The study involved three phases: In the first phase, the female experimenter read a list of 20 words for recall in phase three. For every word correctly recalled, they would earn 50 cents, up to a maximum of 10 euros. This amount differs from the Paternoster experiment, where participants were able to earn 1 US dollar for every word. In the second phase, following the reading of the memory recall words, participants had 8 minutes to fill out an online survey with demographic information and control variables. In the third phase, the experimenter started by demonstrating how to correctly enter the recall words into the online platform. In doing so, she “discovered” that there were four links “erroneously” included on the recall page; these links contained the lists of words to be recalled. In order to not “delay the session”, the experimenter asked participants to proceed with the 5-minute recall session while she left the room to obtain technical assistance. By doing so, she presented an opportunity to cheat on this task and earn money illegitimately.

Just as in the original study, in the experimental condition, when the experimenter left the room, the deviant peer confirmed the possibility for deviance, provided a justification, and was then openly deviant by clicking on all four links and subsequently entering the words on the computer screen, which was visible to all participants. In the control condition, the deviant peer sat at the same computer but said nothing and participated in the study as instructed. Unlike in the original study, in our study, the deviant peer was also female. In both conditions, the experimenter returned after 5 minutes of recall and explained that she no longer had time to count the correct words, so all participants would receive 10 euros (the maximum) as compensation for their time.

## Measures

### Deviancy

Deviancy was measured by the number of links that were clicked on (min = 0 to max = 4). Clicking on the link not only clearly violated the objective of the study but also enabled the participant to illegitimately earn money. This deviancy can, therefore, be considered as cheating but also as petty theft.

### Theoretical control variables

Four control variables were included in this study based on theoretical hypotheses and empirical evidence that has shown them to predict (a lack of) deviancy and delinquency.

### Personality

Conscientiousness, defined as “the tendency to be organized, responsible, and hardworking” (Krueger et al. 1994) was measured using a shortened version of Goldberg’s Big Five Personality Questionnaire (Gerris et al. 1998). This 6-item subscale includes items such as “I am accurate” (1 = completely untrue to 7 = completely true) and was included because delinquency abstainers are thought to have (personal) characteristics that aid them in avoiding delinquency (Moffitt 1993). Reliability was very good (α = 0.90).

### Parental support

Parental support was measured using the 6-item support subscale of the Network of Relationships Inventory (Furman and Buhrmester 1985). The scale includes items such as “To what extent does your parent help you figure out or fix things?” (1 = little to not at all and 5 = as much as possible). Reliability was very good (α = 0.90). This measure was included because bonds with parents are thought to prevent delinquency (Hirschi 1969).^1^

### Importance of peers and school achievement

Additionally, following Paternoster et al. (2013), we also asked participants how important friends and school were to them, which may be related to both cheating and vulnerability to deviant peer exposure (Hirschi 1969; Moffitt 1993). “How important is it to you that you have lots of friends?” (1 = not at all important and 5 =very important) and “how important is it to you that you do well in school?”

## Results

Table 1 shows that, overall, the experimental and control conditions were very similar in regards to theoretical and demographic variables; the only significant difference was that there were only native Dutch participants in the experimental condition, whereas the control condition was comprised of both native Dutch and non-native Dutch participants (*n* = 6).

**Table 1:** Descriptive statistics for the total sample and two conditions

	Total sample, *n* = 69		Control, *n* = 36		Experimental, *n* = 33		*t*
	M	SD	M	SD	M	SD	
Female	0.61	0.49	0.58	0.50	0.64	0.49	− 0.445
Age	20.64	2.00	20.39	1.66	20.91	2.31	− 1.066
Dutch	0.91	0.28	0.83	0.37	1.00	0.00	− 2.65*
Importance of school	4.13	0.54	4.11	0.58	4.15	0.51	− 0.308
Importance of friends	3.55	0.68	3.56	0.61	3.55	0.75	0.062
Conscientiousness	4.71	1.16	4.78	1.05	4.63	1.27	0.521
Support	3.60	0.68	3.66	0.69	3.55	0.68	0.647

***p < 0.001, **p < 0.01, *p < 0.05

## Deviancy

A Chi-square test was used to examine the bivariate relation between condition and a dichotomous measure of cheating (yes/no). In our data, this relation was non-significant χ^2^ (1,* N* = 69) = 2.47, *p* = 0.12. Overall, 28% of participants engaged in deviancy by clicking on one or more links in the online experiment. However, unlike in the experiment of Paternoster et al. (2013), deviancy was not limited to those who were exposed to a deviant peer. In the control condition, 7 out of 36 participants (19%) clicked on one or more links. Of the 33 participants exposed to a deviant peer, 12 (36%) engaged in deviancy by clicking on one or more links.

Additionally, we conducted additional zero-inflated Poisson (ZIP) regression analyses so that we could examine if being exposed to a deviant peer would predict cheating versus not cheating, but also to examine if, once cheating has occurred, exposure to a deviant peer would predict the amount of cheating.^2^ We used cluster-robust standard errors in order to account for any non-independence of observations introduced by collecting data in six sessions. Table 2 presents the results of the ZIP regression analyses. Model 1 shows that, just as in the Chi-square analyses, exposure to a deviant peer did not predict who would be non-deviant (zero-inflation parameter, likelihood of clicking on zero links) but did predict an increase in the total number of links clicked. Model 2 shows that these results remain the same after the addition of control variables related to delinquency.^3^ Based on the combination of our Chi-square results as well as our more conservative additional analyses, we can conclude that exposure to a deviant peer did not predict who would be deviant versus non-deviant, but did increase the amount of deviancy amongst participants who cheated. Additional analyses regarding the number of words claimed to be correctly recalled can be found in the Technical Appendix.

**Table 2:** Zero-inflated Poisson (ZIP) regressions for deviant peer exposure predicting the probability of being a non-cheater and the amount of deviancy

	Model 1						Model 2					
	Non-cheater			Amount of deviancy			Non-cheater			Amount of deviancy		
	B	SE B	OR	B	SE B	IRR	B	SE B	OR	B	SE B	IRR
Deviant peer exposure	− 0.50	0.55	0.61	0.82*	0.40	2.28	− 0.50	1.03	0.61	1.19**	0.35	3.30
Female							− 0.62	1.46	0.54	− 0.57	0.58	0.57
Age							0.07	0.18	1.08	− 0.02	0.06	0.98
Importance of school							− 0.80	1.02	0.45	0.45	0.35	1.57
Importance of friends							− 0.78	2.18	0.46	− 0.53	0.47	0.59
Conscientiousness							− 0.02	0.75	0.99	− 0.17	0.33	0.84
Support							− 0.82	0.56	0.44	0.10	0.23	1.11

***p < 0.001, **p < 0.01, *p < 0.05. IRR = incidence rate ratio. OR = odds ratio. Deviant peer exposure was the experimental condition. Amount of deviancy is the number of links clicked. The probability of being a non-cheater is the zero-inflated parameter predicting a zero score

## Discussion

In this study, we replicated previous work (Paternoster et al. 2013) showing that a brief exposure to a deviant peer increased the amount of deviancy in which participants engaged. However, unlike the original study, we did not find that only participants exposed to a deviant peer engaged in deviancy. Nevertheless, this replication study further generalizes this design and its conclusions regarding the influence of a deviant peer on the amount of deviancy to additional conditions and populations.

While the results of our study were more modest than those in the original study (Paternoster et al. 2013), these cannot be attributed to the use of a female deviant peer, as the percentage of people who cheated after exposure to the deviant peer were highly comparable between both studies (36% vs. 38% in Paternoster et al. 2013). Rather, we think that the use of a female deviant peer was a strength of this replication study. By varying the gender of the deviant peer from the original experiment, we were able to further generalize the nature and strength of deviant peer influence.

Instead, the differences between our studies were the result of people also cheating in the control condition. While we do not have a definite answer for why some participants in the control condition cheated in our study but not in the study of Paternoster et al. 2013 nor in Gallupe et al. 2016, these results are not theoretically implausible. Peer deviancy is a strong predictor of own delinquency; however, it is not the only predictor of own delinquency (e.g., Pratt et al. 2010). Further, the opportunity to cheat in both conditions was inherent to the study design. A randomized design does not preclude that some predictors of delinquency may be exacerbated by the presence of a deviant peer, and, therefore, may be related to both deviancy in the control condition and to susceptibility to deviant peer influence increasing the amount of deviancy after exposure to a deviant peer. Therefore, future research should employ a similar experimental design, in a much larger sample size, to also examine factors that might increase the susceptibility to deviant peers.

Moreover, given that the conditions of this experiment differ from practices described in social learning or differential association theory (e.g., are static and immediate vs. dynamic and occurring over time), it is of particular importance to consider the situational factors that may have played a role in explaining our findings. One of the situational or methodological explanations for why this difference between studies may have occurred is that a female experimenter conducted our study. While we are not sure exactly why this would impact cheating in the control condition, it could well be that specific characteristics of the experimenter may play an additional role in explaining deviant behavior (e.g., Farrington and Kidd 1977). For example, these characteristics may be related to any cost–benefit calculations in situational risky decision-making (e.g., likelihood of detection).

Second, participants may have decided that there was a threshold of money that would be worth their time or risk. For example, the average number of words recalled by a non-cheater would have earned them between 3 and 4 euros.^4^ Therefore, participants in both conditions may have felt more entitled to cheat in order to earn additional money for their time. Alternatively, we could also hypothesize that, because the reward for deviancy was lower in our study, motivation to cheat may also be lower. Therefore, we cannot be certain in which way the monetary value per word impacted the results. Previous research suggests that, when differences between monetary rewards for deviance are small (e.g., 50 p vs. 1 £), the amount does not impact the occurrence of deviancy, though when differences are larger (e.g., 1 £ vs. 5 £), deviancy was more likely when rewards were higher (Farrington and Knight 1979). However, none of this work considered the additional influence of deviant peers. Research should include conditions that vary only in their reward for deviance and also examine the potential different thresholds at which peer deviancy does not have an influence on own deviancy (e.g., Miller 2010). Furthermore, future research should consider to what extent participants’ own economic conditions and the strength of their financial motivations for participating in the study may have played a role. While household economic conditions are not related to minor offending (Bjerk 2007), perceptions of financial difficulties are. Hoeve et al. (2016) found that financial problems are linked to delinquency, and over 50% of students in the Netherlands perceive their financial situation to be reasonable to very bad (van den Broek et al. 2011). Therefore, participants’ own situational financial incentives may be relevant as well.

Finally, in this study, we examined the exposure to one deviant peer. Research should also consider different combinations of deviancy that might better reflect a naturalistic peer group. For instance, what would happen to deviant peer influence in situations where peer deviancy is inconsistent (pro- and antisocial group members) versus overwhelmingly deviant (e.g., Asch 1956; Milgram 1963) and how do these different deviant peer combinations impact the threshold of deviancy to which participants allow themselves to be influenced? Indeed, recent experimental work has already found that theft of a gift card increased when two deviant peer confederates were present compared to only one (Gallupe et al. 2016).

## Conclusion

In conclusion, this study replicates the finding that a brief exposure to deviant modeling by a previously unknown peer subsequently increases the *amount of deviant* behavior in young adults. However, we did not replicate the finding that only participants who were exposed to a deviant peer engaged in deviancy. Future research should try to experimentally examine delinquency predictors that are particularly susceptible to being exacerbated by the influence of a deviant peer as well as plausible thresholds for the influence of deviant peer modeling on own deviancy.

## Electronic supplementary material

Below is the link to the electronic supplementary material.

ESM 1 (DOCX 17.6 kb)

## Footnotes

1 Parental support may be expected to have less of an influence in young adults as opposed to (younger) adolescents, but is, nevertheless, an important theoretical predictor in line with social control theory.

2 The results do not differ if data are analyzed with separate logistic and OLS regressions.

3 Ethnicity is not included as a control variable, as every non-native Dutch participant was a non-cheater, producing computational errors. The results did not change when non-native Dutch participants were removed from the analyses.

4 Earning 3–5 euros per 30 minutes is consistent with the standard for participating in social science research at the university.
