# Supplementary material for: An experimental investigation of the influence of deviant peers on own deviancy: A replication study
Source: J Exp Criminol. 2017 Oct 1;14(3):429–38. doi: 10.1007/s11292-017-9305-3 (PMC6417371; doi:10.1007/s11292-017-9305-3)
Supplement: Supplementary file 1 — (DOCX 17.6 kb) [file 11292_2017_9305_MOESM1_ESM.docx]

**Technical Appendix**

**Detailed Procedure**
 This procedure is a replication of Paternoster et al. (2013). The online survey was programmed in UniPark (Questback) software. The study involved three phases: In the first phase, the experimenter read a list of 20 words which participants were asked to recall at the end of the experiment. The experimenter informed participants that for every word they remember correctly they would earn 50 cents to a maximum of 10 euro. In the second phase, following the reading of the memory recall words, participants were asked to fill out an online survey with demographic information and control variables. Additionally, in line with the premise of a memory recall study participants were asked two questions about their perceived memory ability. These questions were: “In general, I have a good short-term memory” and “In general, I have a good long-term memory” (1 = *completely disagree* to 5 = *completely agree*). Participants had 8 minutes to fill out the survey questions. They were asked to wait for further instruction from the experimenter before proceeding to the next screen so that all participants would have the same amount of recall time. In the third phase, after the 8 minutes had passed, the experimenter then demonstrated how to correctly enter the recall words into the online platform. First, the experimenter asked all participants to click on the survey link to “enter recall words” in the online survey. The experimenter then acted surprised to see that there were four random links on the recall page of the survey. The experimenter clicked on the links on her computer and announced that these links were erroneously included on the recall page, as they contained the lists of words to be recalled. She then checked to confirm whether participants also had these erroneous links in their survey. Upon finding that they did, the experimenter then announced that this was a software bug – and reminded participants that the purpose of the experiment is to recall as many words as possible from memory and not to use the links. The experimenter then left the room to find someone who could assist with removing the links before the next session.
 In order to not “delay the session,” the experimenter asked participants to proceed with the 5-minute recall session while she was gone. By doing so, she presented an opportunity to cheat on this task and earn money illegitimately. In the experimental condition, when the experimenter left the room the deviant peer confirmed the possibility for deviance, provided a justification, and then was openly deviant. The following script was used:
 “That lady was right, you can see the words if you click on the links. I’m going to use them. I thought we were guaranteed 10 euros and not that it would be 50 cents for each word, so I’m using the links.”

The deviant peer then openly cheated by clicking on all four links and subsequently entering the words on the computer screen. In the control condition the deviant peer sat at the same computer but said nothing and participated in the study as instructed. Finally, in both conditions, after the 5-minute recall period ended, the experimenter returned and explained that because of the problems with the software and links she no longer had time to count the correct words, so all participants will receive 10 euro as compensation for their time. The total experiment took approximately 20-25 minutes.

Participants received a debriefing e-mail one week after the study was conducted. They were informed of the true purpose of the study, as well as the possibility to consult the researchers for any questions or concerns. None of the participants contacted the researchers.

**Extra Analyses**

Following Paternoster et al. (2013), we examined if the number of words correctly recalled was related to the number of links clicked in order to test whether clicking on the links was actually representative of deviancy and not just curiosity. Indeed, the number of links clicked significantly predicted the number of words correctly recalled after controlling for all demographic and theoretical variables (β = .77; *p* <.001). Further, participants exposed to a deviant peer recalled significantly more words (*M* = 9.21; *SD* = 4.65), than those in the control condition (*M* = 7.31; *SD* = 2.36), *t*(1, 46.58*)* = -2.12, *p* <.05*.* Additionally, of those who actually clicked on the links, participants in the control condition recalled significantly fewer words (*M* = 8.29; *SD* = 3.15) compared to those who cheated in the experimental condition (*M* = 13.75; *SD* = 4.27), *t*(1, 17*)* = -2.94*, p* <.01. Therefore, it seems that participants exposed to a deviant peer were more purposeful in their intent to click on (more) links in order to illegitimately earn more money (i.e., increase the *amount of* deviancy) than cheaters in the control condition.
